# Transcription-Independent Heritability of Induced Histone Modifications in the Mouse Preimplantation Embryo

**DOI:** 10.1371/journal.pone.0006086

**Published:** 2009-06-30

**Authors:** Matthew D. VerMilyea, Laura P. O'Neill, Bryan M. Turner

**Affiliations:** Chromatin and Gene Expression Group, Institute of Biomedical Research, University of Birmingham Medical School, Birmingham, United Kingdom; Buck Institute for Age Research, United States of America

## Abstract

Enzyme-catalyzed, post-translational modifications of core histones have been implicated in the complex changes in gene expression that drive early mammalian development. However, until recently the small number of cells available from the preimplantation embryo itself has prevented quantitative analysis of histone modifications at key regulator genes. The possible involvement of histone modifications in the embryo's response to extracellular signals, or as determinants of cell fate or lineage progression, remains unclear. Here we describe the use of a recently-developed chromatin immunoprecipitation technique (CChIP) to assay histone modification levels at key regulator genes (*Pou5f1*, *Nanog*, *Cdx2*, *Hoxb1*, *Hoxb9*) as mouse embryos progress from 8-cell to blastocyst in culture. Only by the blastocyst stage, when the embryonic (Inner Cell Mass) and extra-embryonic (Trophoblast) lineages are compared, do we see the expected association between histone modifications previously linked to active and silent chromatin, and transcriptional state. To explore responses to an environmental signal, we exposed embryos to the histone deacetylase inhibitor, anti-epileptic and known teratogen valproic acid (VPA), during progression from 8-cell to morula stage. Such treatment increased H4 acetylation and H3 lysine 4 methylation at the promoters of *Hoxb1* and *Hoxb9*, but not the promoters of *Pou5f1*, *Nanog,Cdx2* or the housekeeping gene *Gapdh*. Despite the absence of detectable *Hoxb* transcription, these VPA-induced changes were heritable, following removal of the inhibitor, at least until the blastocyst stage. The selective hyperacetylation of *Hoxb* promoters in response to a histone deacetylase inhibitor, suggests that *Hox* genes have a higher turnover of histone acetates than other genes in the preimplantation embryo. To explain the heritability, through mitosis, of VPA-induced changes in histone modification at *Hoxb* promoters, we describe how an epigenetic feed-forward loop, based on cross-talk between H3 acetylation and H3K4 methylation, might generate a persistently increased steady-state level of histone acetylation in response to a transient signal.

## Introduction

Specific post-translational modifications of core histones are a central component in the complex network of epigenetic mechanisms by which genes are activated and silenced [1]. Modifications are put in place and maintained by the balanced activity of families of modifying and demodifying enzymes, the activities of which are influenced by the actions of metabolites and a variety of environmental chemicals [2]. These enzymes, and the modifications they put in place, provide a potential interface through which the environment interacts with the genome [3]. It has also been suggested that specific combinations of histone modifications may constitute a code that determines the transcriptional state of chromatin [4], [5], [6]. The code operates, in part, by providing, on the nucleosome surface, an array of modifications that are recognised, individually or in combination, by non-histone proteins that, in turn, exert functional effects. The existence of such interactions and their involvement in ongoing chromatin functions is no longer in doubt and proteins that bind selectively with modified histones, often with chromatin modifying activities, are being identified in growing numbers [7]. However, despite this it remains uncertain whether histone modifications can operate as a true code, able to determine and predict future transcriptional states [8], [9]. A histone code could play a key role in regulating patterns of gene expression through development, but two conditions must be met for this to occur. First, specific histone modifications must be predictive of transcription, even if not directly causative, rather than just reflecting transcriptional states determined by other epigenetic factors. Second, histone modifications induced by developmental or environmental cues and with potential coding roles, must be heritable from one cell generation to the next, in the absence of induced transcriptional change. Both these conditions remain to be demonstrated.

Changes in histone modification seem to be closely involved in the complex changes in gene expression that drive early development [10]. Turnover of acetate groups on bulk histones in mouse embryos has been demonstrated as early as the 1–2 cell stage [11] and microscopical approaches have revealed fascinating inter-blastomere variation in global histone modification levels during the cleavage stages [12], [13]. However, the great majority of bulk histones analysed by western blotting or microscopy, are derived from intergenic chromatin and the patterns of modification observed will not necessarily reflect those present across individual genes. If we are to understand the mechanisms that underpin developmental decisions made during early embryogenesis, it is essential to explore the detailed epigenetic properties of individual genes in the early embryo itself, where cells are present in their normal developmental niche. Until recently, such analysis has not been possible, largely because the small number of cells obtainable has precluded the use of Chromatin ImmunoPrecipitation (ChIP), the only experimental approach capable of providing such information. To address this, we recently developed a variant of ChIP (CChIP) that uses “carrier” chromatin from *Drosophila* cells to allow immunoprecipitation of chromatin from as few as 100 mammalian cells [14]. We showed that this approach can quantify histone modifications across key regulator genes such as *Nanog* and *Cdx2* in the mouse blastocyst [14].

Here we use CChIP to define patterns of histone modification at key genes as mouse embryos progress from the 2-cell to blastocyst stage and to show how this pattern can be changed by an environmental agent, namely the deacetylase inhibitor and teratogen valproic acid (VPA) [15], [16], [17]. We find that VPA treatment at the 8-cell to morula stage exerts gene-specific effects, with increased histone acetylation and H3K4 methylation at the transcriptionally silent *Hoxb1* and *Hoxb9* promoters, but little or no effect at the promoters of other genes, both active and silent. Crucially, the VPA-induced changes in histone modification are transmitted, in the absence of inhibitor or of detectable transcription, through to the blastocyst stage, thereby meeting one of the conditions necessary for a predictive histone code.

## Results

### Valproate slows development and selectively alters gene expression

Valproic acid (VPA, usually used as the sodium salt), like other short chain fatty acids, is a broad spectrum inhibitor of both class I and class II histone deacetylases (HDACs) and causes rapid hyperacetylation of bulk histones when applied to cultured cell lines [17]. In common with other HDAC inhibitors, it also leads to increased mono-, di- and tri-methylation of H3 lysine 4, but not of other methylatable histone lysines [18], [19]. This global change may reflect the preferential methylation of hyperacetylated H3 by SET-domain methyltransferases, demonstrated by in-vitro assays [18], or the physical and functional association of histone deacetylases and the demethylase KDM1/LSD1 [20]. Although the effects of VPA on bulk histone modifications are well defined, the extent to which they are reflected in altered histone modifications at control regions of defined genes, has not so far been reported.

**Figure 1:**
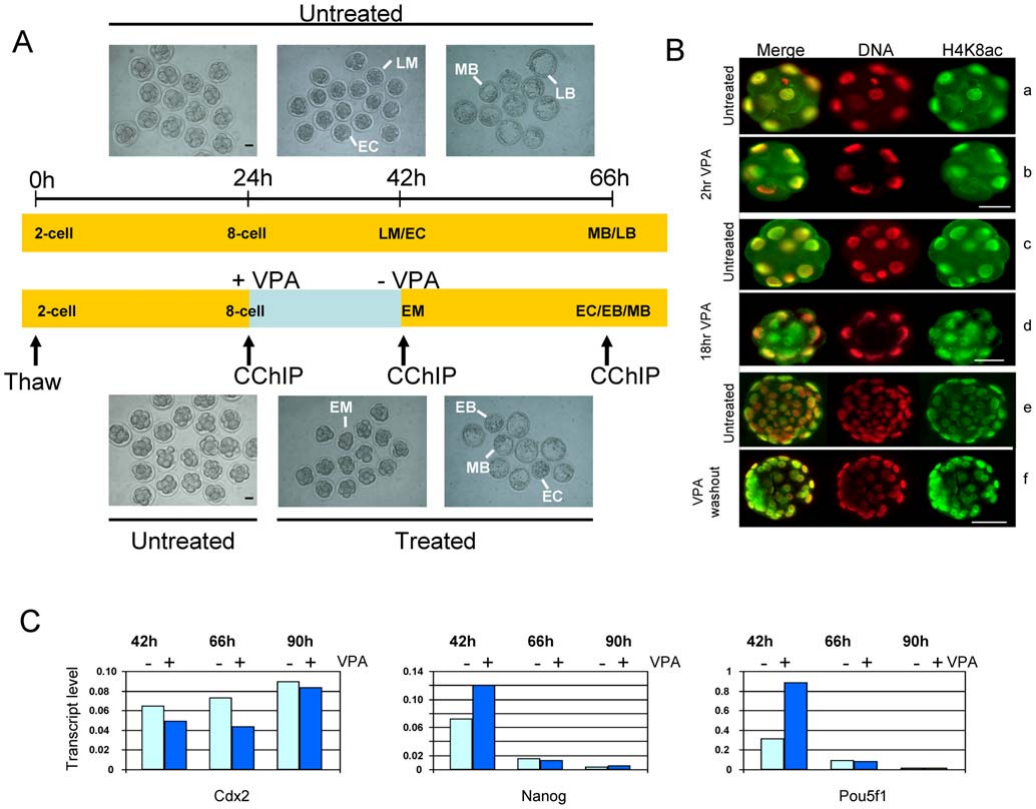
Effects of valproic acid (VPA) on developmental progression, morphology and expression of key regulator genes in cultured mouse embryos. A. Cryopreserved 2-cell *Balb/c* mouse embryos were thawed and cultured with or without 1 mM sodium valproate (VPA) as shown. Pooled embryos were harvested for CChIP at the times indicated. Photographs show representative examples of embryos at the stages indicated. EM =  early morula, LM = late morula, EC = early cavitating blastocyst, EB = early blastocyst, MB = mid-expanded blastocyst, LB = late-expanded blastocyst. Bar = 40 µm. B. Intranuclear distribution of acetylated histone H4 in 8-cell, morula and blastocyst stage embryos treated or untreated with 1 mM VPA at the 8-cell to morula stage. C. Effect of VPA treatment on expression of *Cdx2*, *Nanog* and *Pou5f1*, relative to *ActB*, in cultured embryos assayed by RTQ-PCR. Cryopreserved 2-cell embryos were grown to the morula (42 h), blastocyst (66 h) or hatching blastocyst (90 h) either untreated (VPA -) or after growth for 18 h in 1 mM VPA from the 8-cell to morula stage (+ VPA). In this series of experiments, embryos were grown for longer than usual (up to 90 h, see text). VPA treatment enhanced expression of *Nanog* and *Pou5f1* at the morula stage (42 h), but this was lost by the blastocyst stage, with a fall of several-fold in transcript levels in both treated and untreated embryos. *Cdx2* expression was unaffected by VPA and showed a slight progressive increase as embryos progressed from morula to hatching blastocyst.

The experimental design used to monitor changes in histone modification in the developing pre-implantation embryo in the presence and absence of VPA, is shown in Figure 1A. Mouse 2-cell embryos were grown in culture for 24 h, at which stage 8-cell embryos were separated into groups of about 50 and further cultured for 18 h (ie. at least one complete cell cycle) in medium with or without 1 mM VPA. In cultured cell lines, VPA slows cell cycle progression and increases apoptosis [17]. In treated embryos, progression to the blastocyst stage was slowed, but we detected no increase in gross morphological defects (Fig. 1A and data not shown). After 18 h the great majority of embryos in the treated group developed to the morula stage, while embryos in the untreated groups had reached the morula or early cavitating blastocyst stage (Table 1, Fig. 1A). Some treated and untreated embryos were immediately harvested for CChIP, while others were washed several times and cultured for an additional 24 h in medium without VPA. At this stage nearly all untreated embryos had reached the mid- to late- (expanded) blastocyst stage while progression of the VPA- treated embryos was relatively retarded, with a higher frequency of cavitating and early blastocysts (Table 1, Fig. 1A). Immunofluorescence labelling with antibodies to acetylated H4 showed essentially uniform staining of nuclei in the 8-cell, morula and blastocyst, with no detectable distortion of either nuclear morphology or the largely uniform intranuclear distribution of acetylated histone (Fig. 1B).

**Table 1:** Effect of valproic acid at the 8-cell to morula stage (24–42 h) on developmental progression of mouse embryos grown from the 2-cell stage in culture.

Treatment, time in culture	Stage				
	Total started	Morula	Cavitating	Early blast.	Mid/Late blast.
Untreated 42 h	140	116 (83%)	24 (17%)	0	0
+VPA 24–42 h	140	126 (90%)	0	0	0
Untreated 66 h	89	0	0	0	86 (97%)
+VPA 24–42 h; -VPA 42–66 h	78	0	15 (19%)	63 (81%)	0

Stages were determined by morphology, as illustrated in Fig. 1A.

Even with pooled embryos, the number of cells available inevitably restricts the number of histone modifications and the number of genes that can be tested by CChIP [14]. We chose to assay three modifications, namely H4 acetylated at lysine 8 (H4K8ac) and H3 tri-methylated at lysine 4 (H3K4me3), modifications associated with transcriptionally active promoters, and H3 di-methylated at lysine 9 (H3K9me2), generally associated with transcriptionally silent promoters [1]. We first tested three key regulator genes, the pluripotency genes *Pou5f1* and *Nanog*, and *Cdx2*, a gene essential for trophectoderm formation [21], [22], [23]. The three genes show very different expression patterns. Microscopical analysis of the protein products [24] indicates that *Pou5f1* is expressed strongly and uniformly in all cells at the 8-cell stage and beyond, while *Nanog* and *Cdx2* are expressed relatively weakly (*Nanog*) or not at all (*Cdx2*) at the 8-cell stage and in some cells, but not others, in the morula. We used RTQ-PCR to confirm expression of *Pou5f1* and *Nanog* at the 8-cell to morula stage and to show that expression was moderately enhanced in VPA-treated morula-stage embryos (Fig. 1C). Enhanced expression was lost following growth to the blastocyst stage in the absence of inhibitor, though by this stage overall expression had fallen several-fold, presumably due to silencing of these two genes in the trophectoderm, the most numerous cell population in the blastocyst (Fig. 1C). In contrast, *Cdx2* expression was modestly reduced by VPA treatment, but rose gradually as development progressed (Fig. 1C).

### Histone modifications at key regulator genes do not reflect transcriptional status prior to the blastocyst stage

**Figure 2:**
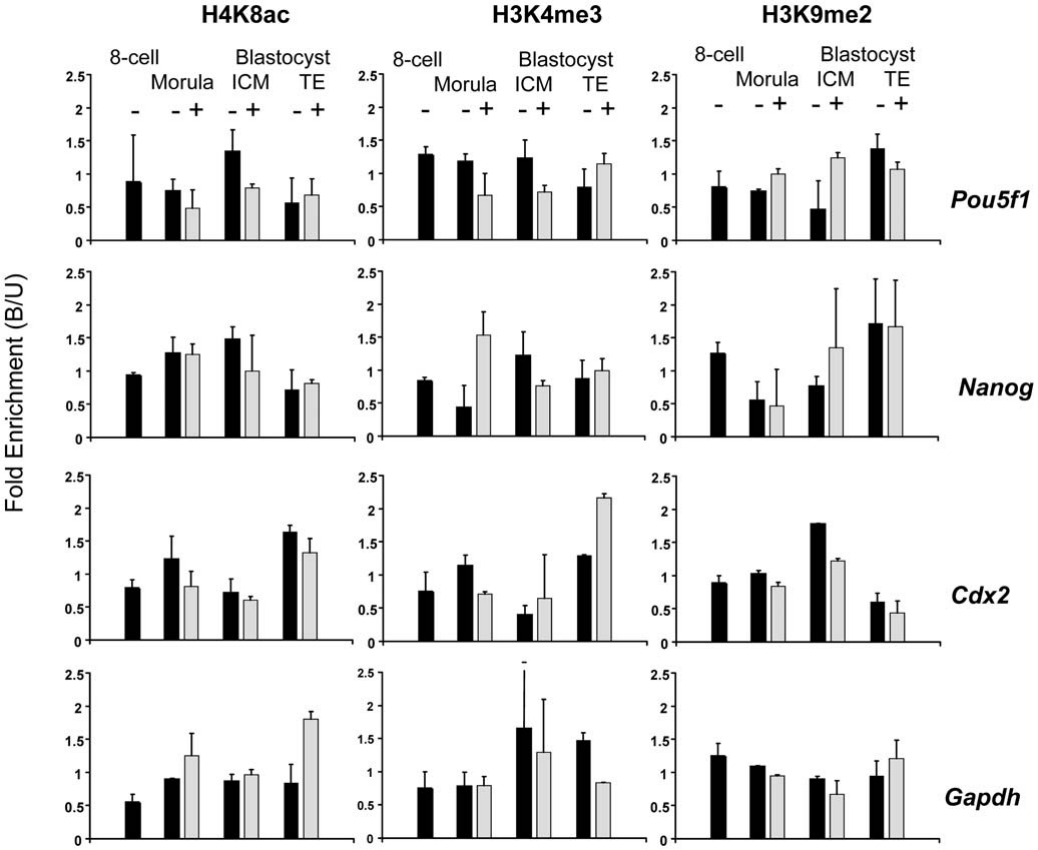
Quantitation by CChIP of levels of histone modification (Bound/Unbound ratio) at selected regions of the Pou5f1, Nanog, Cdx2 and Gapdh promoters. CChIP was carried out as previously described with antibodies to the modifications indicated [14]. Specific sequences were assayed in the precipitated (antibody Bound) and non-precipitated (Unbound) fractions by radioactive PCR [14]. Each column represents the mean from at least two separate experiments calculated, in each experiment, from separate PCR reactions (in duplicate) run for 38 and 41 cycles. Error bars show SE. 8-cell embryos were all untreated (- , dark columns) before CChIP. Morula stage embryos were untreated (-, dark columns) or had been treated with 1 mM VPA for 18 h from the 8-cell stage (+, pale columns). Blastocysts were derived from untreated morulae (-, dark columns) or from morulae grown from the 8-cell stage in 1 mM VPA and then for a further 24 h without VPA (+, pale columns). Inner Cell Mass (ICM) and trophectoderm (TE) were separated as described previously [14].

Levels of H4K8ac, H3K4me3 (often associated with active chromatin) and H3K9me2 (often associated with silent chromatin) at the promoters of *Pou5f1*, *Nanog*, *Cdx2* and *Gapdh* were assayed by CChIP in 8-cell and morula stage embryos. All three modifications show little correlation with transcriptional status (ranging from constitutively active *Gapdh* to silent *Cdx2*) and only modest developmental changes from 8-cell to morula (Fig. 2, compare 8-cell – and Morula -, supplementary Tables S1, S2). Similar results were obtained with antibodies to H4 acetylated at lysine 16 (H4K16ac), again with no correspondence between acetylation and transcriptional state (supplementary Fig. S1). Levels of both H4K16ac and H4K8ac were lower at the promoter of the constitutively active housekeeping gene *Gapdh* than at the promoters of the three lineage specific genes tested (Fig. 2, supplementary Fig. S1).

Because of the lineage-dependent expression patterns of *Pou5f1*, *Nanog* and *Cdx2* [23], [24], we fractionated blastocysts into ICM and trophectoderm. In the ICM, where they are transcriptionally active, the *Pou5f1* and *Nanog* promoters are relatively rich in H4K8ac and H3K4me3, and depleted in H3K9me2, while in the trophectoderm, where they are silent, they are relatively rich in H3K9me2 and depleted in H4K8ac and H3K4me3 (Fig. 2, supplementary Table S3). These findings are consistent with our previous results [14]. The *Cdx2* promoter, active in the trophectoderm and silent in the ICM, shows the opposite pattern of modification, consistent with its transcriptional status (Fig. 2). Thus, levels of histone modification typical of active and silent genes are in place by the blastocyst stage of development, where they reflect transcriptional status. In contrast, at earlier stages the relationship between histone modifications and gene expression levels is less clear cut.

### VPA induces changes in histone modification at Hoxb genes but not at other regulator or housekeeping genes

We detected no significant change in histone acetylation at the promoters of *Cdx2*, *Pou5f1*, *Nanog* and *Gapdh* in embryos treated with VPA from 8-cell to morula-stage (compare Morula – with Morula + in Fig. 2). The only major VPA-induced change at these four genes is an approximately three-fold increase in H3K4me3 at the *Nanog* promoter, a fold-change that reflected primarily the low level of this modification prior to VPA-treatment and that was lost by the blastocyst stage (Fig. 2, supplementary Tables S1, S2). This restricted response to VPA is surprising in view of the global histone hyperacetylation detected in preimplantation embryos and ES cells exposed to HDAC inhibitors [11]. It is however, consistent with the limited transcriptional response to HDAC inhibitors seen in a number of different cell types [25] and with our own recent ChIP data showing that VPA treatment causes little change in acetylation at most promoters tested in both adult and embryonic cultured cells (*Vibhor Gupta, Hannah Stower, BMT, unpublished*). It seems that global changes in histone modification are not necessarily reflected by changes at individual genes.

**Figure 3:**
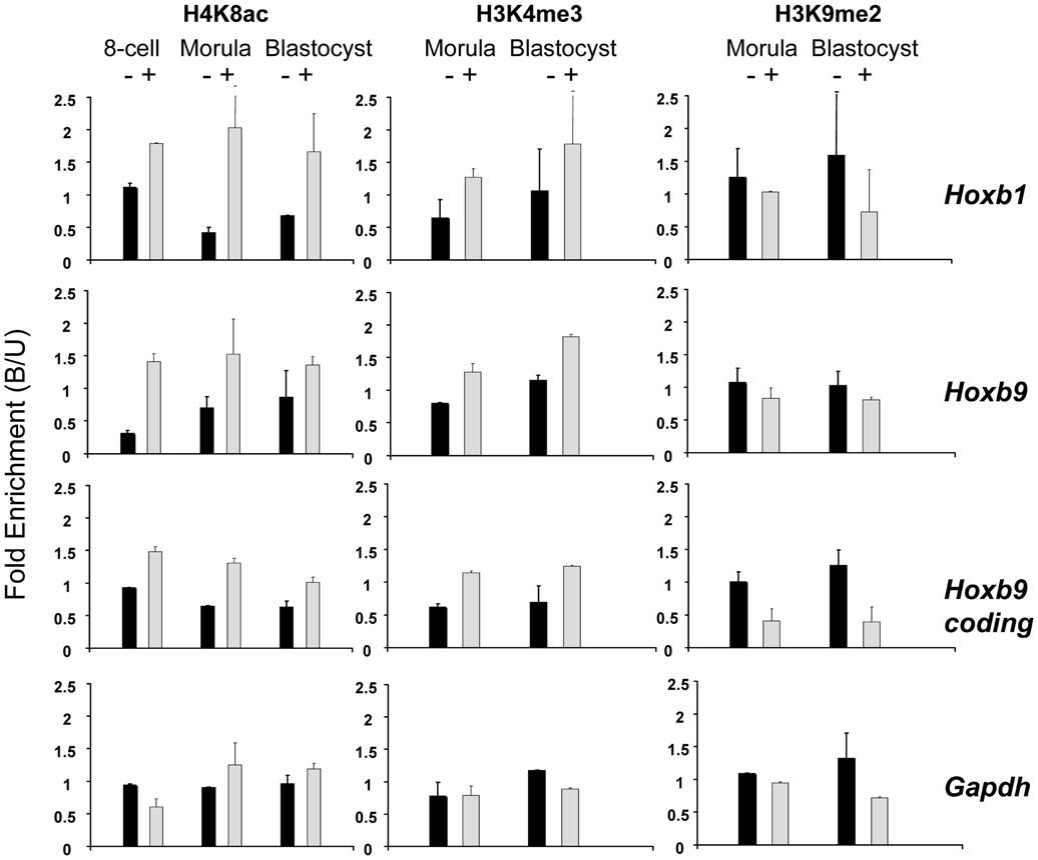
Effects of valproic acid (VPA) on levels of histone modification at Hoxb1, Hoxb9 and Gapdh in the mouse preimplantation embryo. Levels of histone modification (Bound/Unbound ratio) were assayed by CChIP at selected regions of the *Hoxb1* and *Hoxb9* promoters, the *Hoxb9* coding region and the *Gapdh* promoter. 8-cell embryos were untreated (-, dark columns) or treated for 2 h with 1 mM VPA (+, pale columns) before assay by CChIP for H4K8ac. Morula- and blastocyst-stage embryos were untreated (-, dark columns) or treated for 18 h with 1 mM VPA (+, pale columns) as in Fig. 1A before CChIP.

HDAC inhibitors, including VPA, have been shown to alter *Hox* gene expression in mouse embryos, ES cells [26] and human embryonal carcinoma cells [27] and can increase levels of H3 acetylation at *Hoxb1* control elements in mouse ES cells [28]. It has been suggested that aberrant *Hox* gene expression might account for some of the teratogenic effects of VPA [27]. As shown in Fig. 3, CChIP revealed a 1.5–5 fold increase in H4K8 acetylation and H3K4 tri-methylation at the *Hoxb1* and *Hoxb9* promoters and the *Hoxb9* coding region in morula-stage embryos treated with VPA. The increase was consistent across all three *Hoxb* regions tested (supplementary Table S2) and the difference in modification levels between the VPA-treated and untreated *Hox* genes was highly significant (H4K8ac, *P* = 0.006, H3K4me3, *P* = 0.0008; this and all subsequent comparisons use Student's two-tailed paired t test). In contrast, levels of the silencing mark H3K9me2 were consistently lower in VPA-treated morulae (Fig. 3, supplementary Table S2), though at a lower level of significance (*P* = 0.016). To ask whether the valproate-induced changes were contingent upon progression from 8-cell to morula, we treated a group of 8-cell embryos for just 2 h with 1 mM VPA. Both *Hoxb1* and *Hoxb9* showed increased levels of H4K8 acetylation after this brief treatment, whereas *Gapdh* did not (Fig. 3, supplementary Table S1). It seems that histone acetates are turning over rapidly at *Hoxb* promoters in the 8-cell embryo, despite their transcriptional quiescence.

### VPA-induced changes in histone modification at Hoxb genes are heritable through mitosis by a transcription-independent mechanism

The increased levels of H4 acetylation and H3K4 tri-methylation at *Hoxb1* and *Hoxb9* in VPA-treated morulae were still present in the blastocysts derived from them after 24 h growth in the absence of VPA (Fig. 3, compare Morula + and Blastocyst +). At each of the three *Hoxb* regions tested, for both H4K8ac and H3K4me3 and for both replicates, the level of the modification was always higher in blastocysts derived from VPA-treated morulae then from controls (Fig. 3 and supplementary Table S4). The difference between blastocysts derived from VPA-treated and control morulae in modification levels at *Hoxb* regions, was significant (H4K8ac *P* = 0.018, H3K4me3 *P* = 0.00013). There is an indication that the lower levels of H3K9me2 following VPA treatment at the 8-cell to morula stage may also carry through to the blastocyst (Fig. 3, supplementary Table S4), though the statistical significance is borderline (*P* = 0.04). The retention of elevated levels of H4K8ac and H3K4me3 at *Hox* gene promoters following removal of VPA contrasts with the behaviour of bulk histones in cultured cell lines, where VPA-induced increases in both modifications were lost within minutes of removal of the inhibitor [18] (*and unpublished results*).

For the non-*Hox* genes tested in blastocysts, levels of H4K8ac, H3K4me3 and H3K9me2 generally showed little change as a result of VPA treatment at the 8-cell to morula stage (Fig. 2). However, we note that levels of H4K8ac and H3K4me3 at promoters of the pluripotency genes *Nanog* and *Pou5f1*, are consistently *lower* in VPA-treated ICM (where they are active) than in controls, while levels of H3K9me2 tend to be higher (supplementary Table S3). Such changes are not seen in trophectoderm, where these genes are silent, nor at promoters of *Gapdh* or *Cdx2* in either trophectoderm or ICM. These gene- and lineage-specific epigenetic changes may result from the toxic effects of VPA on growth or transcription rate.

Neither the VPA-induced changes in histone modifications at *Hox* gene promoters, nor their heritability through mitosis, are attributable to induced transcription. In both VPA-treated and untreated embryos, transcription was undetectable for *Hoxb9* and barely detectable, even at high cycle numbers with radioactive PCR, for *Hoxb1* (Supplementary Fig. S2). In fact, we detected no induced *Hoxb* expression in embryos transiently exposed to VPA, even when grown to the hatching blastocyst stage, 90 h after initiation of culture (*results not shown*).

## Discussion

The ability of histone deacetylase inhibitor to induce a mitotically heritable change in histone acetylation and gene expression was first demonstrated in the yeast *S.pombe* [29]. Growth for several cell cycles in the presence of the HDAC inhibitor Trichostatin A (TSA) induced hyperacetylation and transcription in normally silent test genes inserted into centric heterochromatin. The active, hyperacetylated state, though reversible at low frequency, was retained through many cell cycles in the absence of inhibitor. However, because acetylation and transcription remained closely linked throughout these experiments, it was not possible to determine which of these two factors was the primary determinant of heritability [29]. More recently, nuclear transplantation in *Xenopus* has been used to show that some genes (eg. the endodermal gene *edd*) can retain a memory of an active gene state, even in an inappropriate (eg. non-endodermal) cell lineage [30]. The memory can be transmitted through up to 24 cell generations from zygote to tadpole. What makes this particularly significant is that through the first 12 cleavage divisions of the *Xenopus* embryo, there is no genomic transcription, so the memory mechanism involved does not require active transcription. Chromatin seems to have a role in this memory in that the variant histone H3.3 and specifically its methylatable lysine 4 residue, seems to be necessary for re-expression (memory) of the active state after progression through the early cleavage cycles [30]. H3.3 associates preferentially with active genes [31], [32] and may play a role in the maintenance of an active state, even in the absence of ongoing transcription.

CChIP has allowed us, for the first time, to compare the expression and chromatin modification patterns of selected genes in the pre-implantation mouse embryo and to study the effects of the histone deacetylase inhibitor and teratogen valproic acid (VPA). There is, amongst the genes studied here, a surprising lack of correlation between transcriptional activity and the levels of histone modifications usually considered to be “activating”, namely H4 acetylation and H3K4 tri-methylation. Thus, VPA increased acetylation levels at the silent *Hoxb1* and *Hoxb9* promoters without any detectable induction of transcription and increased expression of *Nanog* and *Pou5f1* without any parallel increase in acetylation or H3K4 methylation. Similarly, in untreated morula stage embryos, a comparison of active and silent genes reveals no correlation between promoter levels of H4 acetylation and H3K4 tri-methylation and transcriptional status. Only by the blastocyst stage, when genes that are active or silent in the ICM and trophectoderm were compared, did the expected relationships between “active” marks and transcriptional activity become apparent. These findings may be due, at least in part, to differences between cells in gene expression patterns, even in the 8-cell embryo. If, for example, only a minority of blastomeres were to show enhanced *Nanog* expression in response to VPA, increased transcript levels would be readily detected in pooled samples, but increased promoter acetylation, even if it occurs in the minority of expressing cells, would not be.

The increased level of H4 acetylation at *Hoxb* promoters in VPA treated embryos shows that histone acetate groups are turning over, despite the absence of detectable transcription. None of the other genes tested, whether active (*Nanog*, *Gapdh*) or silent (*Cdx2*) showed increased acetylation. This suggests a lack of turnover, though it remains possible that turnover is mediated by deacetylases resistant to inhibition by VPA, perhaps SirT1 or other sirtuins [33]. The turnover of histone acetates at *Hoxb* promoters provides a system through which the promoter chromatin of selected genes can respond to environmental or developmental signals that act on the relevant modifying and demodifying enzymes. Perhaps, in the early embryo high turnover of histone acetates marks genes that are silent but “poised”, ready to be expressed later in development [10]. This silent but poised state contrasts with that of lineage determinants such as *Pou5f1*, *Nanog* and *Cdx2*, where differing expression patterns are already being put in place at the morula-blastocyst transition and earlier. We show that VPA-induced increases in histone acetylation, and contingent H3K4 methylation, at *Hox* gene promoters are not sufficient to override silencing signals and induce transcription. However, these VPA-induced changes are inherited, at least until the blastocyst stage and in the continuing absence of detectable transcription. This finding not only establishes that a change in histone modification level induced by an environmental agent can be heritable, but also provides a process by which the effects of a transient, possibly stage-specific, signal might be transmitted to a later developmental stage without initiating inappropriate transcription [9].

**Figure 4:**
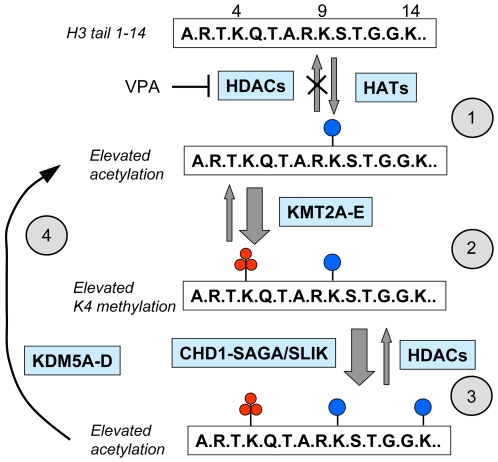
An epigenetic feed-forward loop; a model that links acetylation and lysine 4 methylation on the histone H3 tail and that can maintain elevated levels of histone acetylation. (1) Residues 1–14 of the N-terminal tail of histone H3 are shown (boxed,single letter code), with lysines 4, 9 and 14 indicated. H3 lysines 9,14, 18 and 23 (only the first two are shown) can be acetylated (blue disc) by various histone acetyltransferases (HATs) [2], [41] and deacetylated by various deacetylases (HDACs) [2], mainitaining a dynamic steady state level of acetylation. Vaproic acid (VPA) inhibits most HDACs, thereby increasing acetylation levels. (2) H3 lysine 4 can be tri-methylated (red discs) by SET-domain lysine methyltransferases, specifically KMT2A-E, and demethylated by lysine demethylases, specifically KDM5A-D. Elevated acetylation of the H3 tail increases the H3K4 methylating activity of SET domain methyltransferases [18] (large arrow), leading to enhanced K4 methylation of the acetylated tail domain. (3) CHD1, a component of the SAGA/SLIK histone acetyltransferase complex, binds selectively to H3 tri-methylated at lysine 4 [34], [35]. H3 acetylation is thus further enhanced (large arrow), both through targeting of CHD1-linked acetyltransferase activities and because H3 methylated at lysine 4 is a better substrate for the SAGE/SLIK complex [34], [35]. Demethylation of K4 by KDM5A-D will return the cycle to step 2, but while some acetylation is retained (at lysines 9, 14, 18 or 23), K4 methylation will be rapidly reinstated. The cycle will maintain elevated levels of H3 acetylation, even after removal of the original inhibitor (VPA). In the continuing presence of the necessary acetylating and methylating enzymes, only complete deacetylation of the H3 tail will break the cycle by returning it to step 1. Thus, even a transient exposure to an inhibitor such as VPA could trigger a self-sustaining shift to a higher steady-state level of acetylation and lysine 4 methylation.

In considering the mechanisms by which an induced increase in acetylation could be maintained across cell generations, it is important to remember that the measured level of acetylation represents a dynamic equilibrium between the activities of modifying and demodifying enzymes. Inhibition of deacetylases will shift the equilibrium to a more acetylated level, but following removal of the inhibitor, one would expect the steady state level at gene promoters to return rapidly to its pre-inhibition levels, exactly as seen in assays of bulk acetylation [18]. A possible mechanism is suggested by biochemical studies exploring the cross-talk between acetylation and lysine 4 methylation on the H3 tail. In vitro assays have shown that increased acetylation of the H3 tail domain directly causes increased H3K4 methylation by generating a more effective substrate for SET domain methyltransferases [18], [20]. H3K4me3 is an important mark, selectively recognised by several non-histone proteins, including CHD1, a component of the SAGA- and SLIK-dependent histone acetyltransferase complexes [34], [35]. Thus, an inhibitor-induced increase in histone acetylation, with a consequent increase in H3K4 methylation, could bring in additional acetyltransferases and thereby initiate an increased steady-state level of histone acetylation that could be maintained, even in the absence of inhibitor (Fig. 4). This cycle of events constitutes a self-sustaining mechanism by which an environmentally-induced increase in steady-state histone acetylation at selected genes could be maintained in a dynamic system.

If this model, or something like it, is correct, then the ability to put in place a heritable epigenetic change in response to an environmental agent, or to respond to that agent at all, will depend on the presence of a group of enzymes of defined specificity (Fig. 4), perhaps operating as a complex. Our recent studies in mouse ES cells have shown that all *Hoxb* genes respond to VPA with increased histone acetylation and H3K4 methylation, whereas other genes do not (*unpublished results*). However, in contrast to the situation in the embryo itself, these VPA-induced modifications seem not to be heritable through mitosis (*unpublished results*).The consistent differences between *Hox* genes and others, and between different cell types and developmental stages, may reflect the families of enzymes present at selected genomic regions, something that is amenable to experimental testing.

Valproic acid (VPA) is a widely used preventive treatment for seizures and bipolar disorder [36], but its use during pregnancy is associated with an increased risk of congenital abnormalities, often involving a characteristic mix of central nervous system and craniofacial defects [16]. The persistence of VPA-induced *Hox* gene hyperacetylation, raises the possibility that transient exposure to VPA at the very earliest stages of pregnancy could result in disorganised *Hox* gene expression at later stages, with consequent morphological abnormalities [27]. However, it remains to be shown whether altered histone modifications can drive altered gene expression later in development. We find no evidence for premature expression of *Hoxb1* or *Hoxb9* in VPA treated embryos grown to the hatching blastocyst stage, suggesting that silencing signals still predominate. It will be important to determine, perhaps through re-implantation of VPA-treated embryos, whether the induced changes in *Hox* gene acetylation reported here can alter spatial or temporal patterns of *Hox* gene expression later in development when silencing signals are being overridden.

## Materials and Methods

### Antibodies

Rabbit polyclonal antisera to H4K8ac, H4K16ac and H3K4me3 were raised by immunization with synthetic peptides conjugated to ovalbumin as previously described [37]. Antibodies to H3K9me2 were from Upstate (now Millipore, Billerica, MA, USA). Specificity was assayed by inhibition ELISA for all in-house and commercial antisera and checked by western blotting. For all antisera, cross-reaction with epitopes other than that against which the antiserum was raised was insignificant.

### Embryo culture and manipulation

Cryopreserved 2-cell *Balb/c* mouse embryos (Embryotech, Haverhill, MA, USA) were thawed and cultured in M16 medium (Specialty Media, now Millipore, Billerica, MA, USA) for differing times up to the (expanded) blastocyst stage. Isolation of the inner cell mass (ICM) was by immunosurgery [38], with minor alterations [14]. Isolation of mural trophectodermal cells was performed exactly as described previously [14].

### Carrier Chromatin ImmunoPrecipitation, CChIP

For the CChIP procedure, *Drosophila* SL2 cells, cultured as previously described [14] were pelleted and washed 3x in ice cold PBS, 5 mM sodium butyrate. Cells were resuspended to 5×10^7^ cells/ml and 1 ml aliquots were mixed with a small number (for these experiments, at least 100) of mouse cells derived from pooled 8-cell, morula or blastocyst stage embryos. Chromatin was prepared from the mixed sample and immunoprecipitated exactly as previously described [14]. Selected mouse genes were detected in the immunoprecipitated (bound) and non-precipitated (unbound) fractions by radioactive PCR with mouse-specific primers. All PCR reactions were performed in duplicate with mouse and *Drosophila* DNA controls run in parallel to monitor cross-hybridization [14]. Aliquots were removed after 38 and 41 cycles, loaded onto 5% polyacrylamide gels and electrophoresed. Gels were dried onto filter paper (SpeedGel System, Thermo Fisher Scientific Inc., Waltham, MA, USA) for a minimum of 2 hours. Filters were exposed to a phosphor screen overnight and scanned with a PhosphorImager (Typhoon 9200, Amersham, UK). Examples are shown in supplementary Fig. S3. Intensity values for each PCR product were analysed with Image Quant software (Molecular Dynamics, Sunnyvale, CA, USA). Primer pairs and Tm temperatures used are listed in Supplementary Table S5

### Expression analysis by RTQ-PCR

RNA was prepared from mouse embryos as previously described [39]. Transcript levels of *Pou5f1*, *Nanog*, *Cdx2* and *ActB* were quantified by real time PCR using SYBR Green PCR master mix and an ABI 7900 Detection System (ABI Technologies Inc., Barnsley, UK). The primer sequences are listed in Supplementary Table S6.

### Immunofluorescence Labelling of Embryos

Immunofluorescence labelling of embryos was performed as previously described [40] with modifications. Briefly, zona free embryos were placed in a drop of ice-cold ethanol on Poly-Prep slides (Sigma-Aldrich Co. Ltd, Poole, UK) under a dissecting microscope. KCM buffer (120 m*M* KCl, 20 m*M* NaCl, 10 m*M* Tris-HCl, pH 8, 0.5 m*M* EDTA, 0.1% Triton X-100) was added for 10 minutes at room temperature. Excess KCM was removed with PVA absorption spears (Altomed, Tyne and Wear, UK). 50 µl rabbit antibody in 1% BSA/KCM, was added and incubated in a humid chamber at 4°C for 1 hour. Slides were rinsed in KCM, followed by 50 µl of FITC-conjugated goat anti-rabbit IgG antibody (Sigma) at×50 dilution in 1% BSA/KCM. After 1 hour as above, slides were rinsed, fixed in 4% formaldehyde in KCM for 10 minutes and counterstained with DAPI (1 µg/ml) in Vectorshield (Vector Labs, Burlingame, CA, USA).

## Supporting Information

Figure S1 CChIP analysis of H4K16 acetylation at selected promoters in pooled 8-cell embryos or morulae.
(0.05 MB PDF)

Figure S2 Expression of Hoxb1 and Hoxb9 assayed by radioactive PCR in embryos cultured with or without valproic acid.
(0.06 MB PDF)

Figure S3 Examples of gels resolving 32P-labelled PCR products generated from antibody bound and unbound fractions derived by CChIP.
(0.19 MB PDF)

Table S1 ChIP data; 8-cell
(0.05 MB DOC)

Table S2 ChIP data; morula
(0.05 MB DOC)

Table S3 ChIP data; fractionated blastocyst
(0.08 MB DOC)

Table S4 ChIP data; whole blastocyst
(0.05 MB DOC)

Table S5 Primers used for CChIP analysis
(0.03 MB DOC)

Table S6 Primers used for expression analysis
(0.03 MB DOC)
