# Supplementary material for: Transcription-Independent Heritability of Induced Histone Modifications in the Mouse Preimplantation Embryo
Source: PLoS One. 2009 Jun 30;4(6):e6086. doi: 10.1371/journal.pone.0006086 (PMC2698989; doi:10.1371/journal.pone.0006086)
Supplement: Figure S1 — CChIP analysis of H4K16 acetylation at selected promoters in pooled 8-cell embryos or morulae. (0.05 MB PDF) [file pone.0006086.s001.pdf]

Supplementary Figure S1

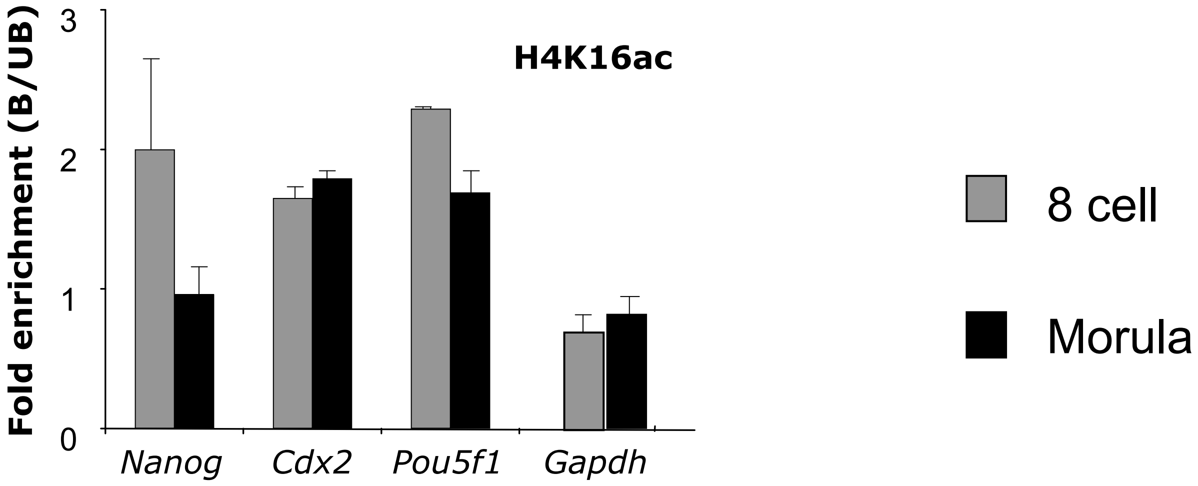

CChIP analysis of levels of H4K16 acetylation at selected promoters in pooled 8-cell embryos (pale bars) or morulae (dark bars)
