# Supplementary material for: Transcription-Independent Heritability of Induced Histone Modifications in the Mouse Preimplantation Embryo
Source: PLoS One. 2009 Jun 30;4(6):e6086. doi: 10.1371/journal.pone.0006086 (PMC2698989; doi:10.1371/journal.pone.0006086)
Supplement: Figure S2 — Expression of Hoxb1 and Hoxb9 assayed by radioactive PCR in embryos cultured with or without valproic acid. (0.06 MB PDF) [file pone.0006086.s002.pdf]

Supplementary Figure S2

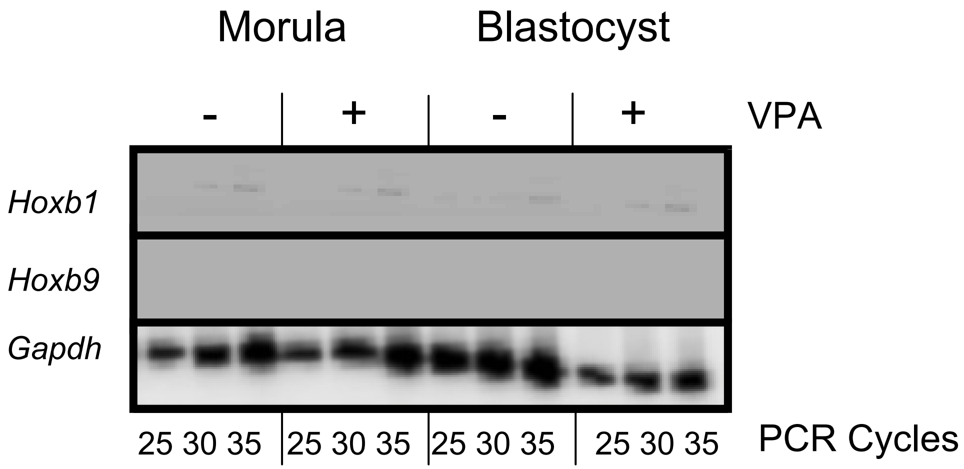

Hox Gene Expression Analysis by radioactive PCR

Expression of *Hoxb1*, *Hoxb9* and *Gapdh* in morula or blastocyst-stage embryos cultured for 18h from the 8-cell to morula stage either with (+) or without (-) 1mM valproic acid (VPA) as indicated. High purity cDNA was obtained from purified mRNA and analysed by radioactive PCR as described by O'Neill, L.P. et al. Nat Genet. 38, 835-841 (2006). Samples were taken at 25, 30 and 35 cycles as indicated.
