# Supplementary material for: Transcription-Independent Heritability of Induced Histone Modifications in the Mouse Preimplantation Embryo
Source: PLoS One. 2009 Jun 30;4(6):e6086. doi: 10.1371/journal.pone.0006086 (PMC2698989; doi:10.1371/journal.pone.0006086)
Supplement: Figure S3 — Examples of gels resolving 32P-labelled PCR products generated from antibody bound and unbound fractions derived by CChIP. (0.19 MB PDF) [file pone.0006086.s003.pdf]

# Supplementary Figure S3

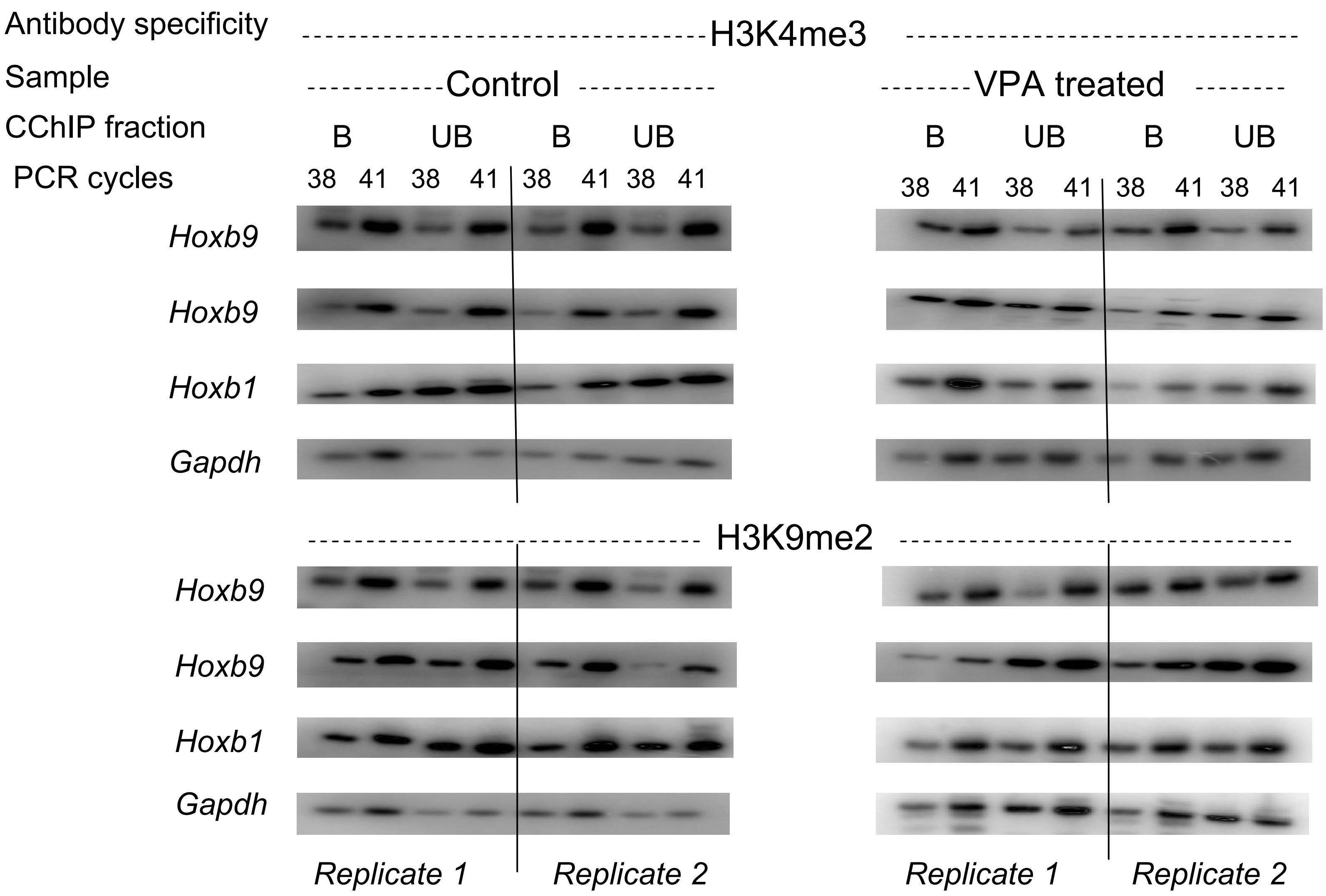


Gels resolving $^{32}P$ labelled PCR products generated from antibody bound (B) and unbound (UB) fractions from CChIP. The products shown are from blastocysts grown from VPA treated or control morulae precipitated with antisera to H3K4me3 or H3K9me2
