# Supplementary material for: Transcription-Independent Heritability of Induced Histone Modifications in the Mouse Preimplantation Embryo
Source: PLoS One. 2009 Jun 30;4(6):e6086. doi: 10.1371/journal.pone.0006086 (PMC2698989; doi:10.1371/journal.pone.0006086)
Supplement: Table S1 — ChIP data; 8-cell (0.05 MB DOC) [file pone.0006086.s004.doc]

**Supplementary Table S1**

Tables S1-S4 show CChIP data on mouse preimplantation embryos

Values shown are B/UB ratios each averaged from 38- and 41-cycle hot PCR duplicates (see supplementary Figure S3)

-/+ indicates untreated and VPA treated (1mM, 18h through 8-cell to morula stage unless otherwise indicated).

**8-cell**

*Modification levels at selected genes in untreated embryos (expt. 1, replicates 1 and 2 as indicated) and effects of 1mM VPA for 2h on H4K8ac at Hoxb genes (expt. 2, +/- VPA as indicated)*

| GENE | 8-cell (expt.1) | | | | | | 8-cell (expt.2) | | | | | |
| --- | --- | --- | --- | --- | --- | --- | --- | --- | --- | --- | --- | --- |
|  | H4K8ac | | H3K4me3 | | H3K9me2 | | H4K8ac | |  | |  | |
|  | 1 | 2 | 1 | 2 | 1 | 2 | - | + |  |  |  |  |
| *Hoxb1* |  |  |  |  |  |  | 1.12 | 1.79 |  |  |  |  |
| *Hoxb9* |  |  |  |  |  |  | 0.31 | 1.41 |  |  |  |  |
| *Hoxb9ex* |  |  |  |  |  |  | 0.93 | 1.48 |  |  |  |  |
| *Gapdh* |  |  |  |  |  |  | 0.95 | 0.61 |  |  |  |  |
|  |  |  |  |  |  |  |  |  |  |  |  |  |
| *Pou5f1* | 0.88 | 0.90 | 1.20 | 1.38 | 0.65 | 0.97 |  |  |  |  |  |  |
| *Nanog* | 0.87 | 1.03 | 0.88 | 0.83 | 1.15 | 1.39 |  |  |  |  |  |  |
| *Cdx2* | 0.83 | 0.78 | 0.96 | 0.57 | 0.97 | 0.84 |  |  |  |  |  |  |
| *Gapdh* | 0.64 | 0.48 | 0.94 | 0.59 | 1.13 | 1.39 |  |  |  |  |  |  |
|  |  |  |  |  |  |  |  |  |  |  |  |  |
