# Supplementary material for: Transcription-Independent Heritability of Induced Histone Modifications in the Mouse Preimplantation Embryo
Source: PLoS One. 2009 Jun 30;4(6):e6086. doi: 10.1371/journal.pone.0006086 (PMC2698989; doi:10.1371/journal.pone.0006086)
Supplement: Table S2 — ChIP data; morula (0.05 MB DOC) [file pone.0006086.s005.doc]

**Supplementary Table S2**

Tables S1-S4 show CChIP data on mouse preimplantation embryos

Values shown are B/UB ratios each averaged from 38- and 41-cycle hot PCR duplicates (see supplementary Figure S3)

-/+ indicates untreated and VPA treated (1mM, 18h through 8-cell to morula stage unless otherwise indicated).

**MORULA**

*Effects of 1mM VPA for 18h (8-cell to morula) on modification levels*

| GENE | REPLICATE 1 | | | | | | REPLICATE 2 | | | | | |
| --- | --- | --- | --- | --- | --- | --- | --- | --- | --- | --- | --- | --- |
|  | H4K8ac | | H3K4me3 | | H3K9me2 | | H4K8ac | | H3K4me3 | | H3K9me2 | |
|  | - | + | - | + | - | + | - | + | - | + | - | + |
| *Hoxb1* | 0.48 | 2.49 | 0.75 | 1.15 | 1.56 | 1.02 | 0.36 | 1.57 | 0.45 | 1.36 | 0.95 | 0.98 |
| *Hoxb9* | 0.59 | 1.13 | 0.81 | 1.37 | 0.94 | 0.72 | 0.82 | 1.89 | 0.79 | 1.20 | 1.23 | 0.94 |
| *Hoxb9ex* | 0.65 | 1.36 | 0.58 | 1.12 | 0.90 | 0.28 | 0.63 | 1.27 | 0.66 | 1.16 | 1.11 | 0.53 |
| *Gapdh* | 0.60 | 1.48 | 0.92 | 1.11 | 1.36 | 0.96 | 0.52 | 1.02 | 0.64 | 1.00 | 1.12 | 0.86 |
|  |  |  |  |  |  |  |  |  |  |  |  |  |
| *Pou5f1* | 0.63 | 0.29 | 1.27 | 0.44 | 0.77 | 1.06 | 0.88 | 0.67 | 1.10 | 0.90 | 0.72 | 0.95 |
| *Nanog* | 1.03 | 1.26 | 0.20 | 1.28 | 0.35 | 0.08 | 1.51 | 1.24 | 0.67 | 1.77 | 0.75 | 0.87 |
| *Cdx2* | 1.41 | 0.92 | 1.02 | 0.68 | 1.00 | 0.79 | 1.06 | 0.70 | 1.25 | 0.63 | 1.06 | 0.88 |
| *Gapdh* | 0.91 | 1.49 | 0.93 | 0.89 | 1.10 | 0.96 | 0.89 | 1.02 | 0.64 | 0.70 | 1.08 | 0.94 |
|  |  |  |  |  |  |  |  |  |  |  |  |  |
