# Supplementary material for: Transcription-Independent Heritability of Induced Histone Modifications in the Mouse Preimplantation Embryo
Source: PLoS One. 2009 Jun 30;4(6):e6086. doi: 10.1371/journal.pone.0006086 (PMC2698989; doi:10.1371/journal.pone.0006086)
Supplement: Table S3 — ChIP data; fractionated blastocyst (0.08 MB DOC) [file pone.0006086.s006.doc]

**Supplementary Table S3**

Tables S1-S4 show CChIP data on mouse preimplantation embryos

Values shown are B/UB ratios each averaged from 38- and 41-cycle hot PCR duplicates (see supplementary Figure S3)

-/+ indicates untreated and VPA treated (1mM, 18h through 8-cell to morula stage unless otherwise indicated).

**FRACTIONATED BLASTOCYST**

-/+ indicates untreated and VPA treated (1mM, 18h through 8-cell to morula stage) then grown for a further 24h without VPA to the blastocyst stage. Blastocysts were fractionated into ICM (by immunosurgery) and trpohectoderm (by dissection) before CChIP.

**Inner Cell Mass (ICM)**

| GENE | REPLICATE 1 | | | | | | REPLICATE 2 | | | | | |
| --- | --- | --- | --- | --- | --- | --- | --- | --- | --- | --- | --- | --- |
|  | H4K8ac | | H3K4me3 | | H3K9me2 | | H4K8ac | | H3K4me3 | | H3K9me2 | |
|  | - | + | - | + | - | + | - | + | - | + | - | + |
| *Hoxb1* |  |  |  |  |  |  |  |  |  |  |  |  |
| *Hoxb9* |  |  |  |  |  |  |  |  |  |  |  |  |
| *Hoxb9ex* |  |  |  |  |  |  |  |  |  |  |  |  |
| *Gapdh* |  |  |  |  |  |  |  |  |  |  |  |  |
|  |  |  |  |  |  |  |  |  |  |  |  |  |
| *Pou5f1* | 1.48 | 0.75 | 1.21 | 0.71 | 0.18 | 1.29 | 1.21 | 0.83 | 1.26 | 0.72 | 0.77 | 1.25 |
| *Nanog* | 1.33 | 1.17 | 0.96 | 0.70 | 0.68 | 0.72 | 1.63 | 0.83 | 1.48 | 0.82 | 0.87 | 1.97 |
| *Cdx2* | 0.71 | 0.61 | 0.50 | 0.31 | 2.04 | 1.21 | 0.74 | 0.58 | 0.31 | 0.98 | 1.53 | 1.23 |
| *Gapdh* | 0.94 | 0.90 | 2.39 | 1.86 | 0.88 | 0.81 | 0.81 | 1.02 | 0.92 | 0.72 | 0.92 | 0.52 |
|  |  |  |  |  |  |  |  |  |  |  |  |  |

**Trophectoderm**

| GENE | REPLICATE 1 | | | | | | REPLICATE 2 | | | | | |
| --- | --- | --- | --- | --- | --- | --- | --- | --- | --- | --- | --- | --- |
|  | H4K8ac | | H3K4me3 | | H3K9me2 | | H4K8ac | | H3K4me3 | | H3K9me2 | |
|  | - | + | - | + | - | + | - | + | - | + | - | + |
| *Hoxb1* |  |  |  |  |  |  |  |  |  |  |  |  |
| *Hoxb9* |  |  |  |  |  |  |  |  |  |  |  |  |
| *Hoxb9ex* |  |  |  |  |  |  |  |  |  |  |  |  |
| *Gapdh* |  |  |  |  |  |  |  |  |  |  |  |  |
|  |  |  |  |  |  |  |  |  |  |  |  |  |
| *Pou5f1* | 0.32 | 0.50 | 0.98 | 1.02 | 1.54 | 0.99 | 0.83 | 0.86 | 0.62 | 1.25 | 1.25 | 1.14 |
| *Nanog* | 0.79 | 0.96 | 1.07 | 1.12 | 2.19 | 1.81 | 0.65 | 0.65 | 0.69 | 0.86 | 1.25 | 1.03 |
| *Cdx2* | 1.42 | 1.28 | 1.27 | 2.22 | 0.50 | 0.32 | 1.85 | 1.36 | 1.31 | 2.12 | 0.69 | 0.56 |
| *Gapdh* | 0.64 | 1.71 | 1.38 | 0.84 | 0.80 | 1.02 | 1.03 | 1.89 | 1.56 | 0.82 | 1.09 | 1.40 |
|  |  |  |  |  |  |  |  |  |  |  |  |  |
