# Supplementary material for: Transcription-Independent Heritability of Induced Histone Modifications in the Mouse Preimplantation Embryo
Source: PLoS One. 2009 Jun 30;4(6):e6086. doi: 10.1371/journal.pone.0006086 (PMC2698989; doi:10.1371/journal.pone.0006086)
Supplement: Table S4 — ChIP data; whole blastocyst (0.05 MB DOC) [file pone.0006086.s007.doc]

**Supplementary Table S4**

Tables S1-S4 show CChIP data on mouse preimplantation embryos

Values shown are B/UB ratios each averaged from 38- and 41-cycle hot PCR duplicates (see supplementary Figure S3)

-/+ indicates untreated and VPA treated (1mM, 18h through 8-cell to morula stage unless otherwise indicated).

**WHOLE BLASTOCYST**

*-/+ indicates untreated and VPA treated (1mM, 18h, 8-cell to morula stage) then grown for a further 24h without VPA to the blastocyst stage. Whole blastocysts were pooled and used for CChIP*

| GENE | REPLICATE 1 | | | | | | REPLICATE 2 | | | | | |
| --- | --- | --- | --- | --- | --- | --- | --- | --- | --- | --- | --- | --- |
|  | H4K8ac | | H3K4me3 | | H3K9me2 | | H4K8ac | | H3K4me3 | | H3K9me2 | |
|  | - | + | - | + | - | + | - | + | - | + | - | + |
| *Hoxb1* | 0.68 | 2.07 | 1.51 | 2.35 | 2.28 | 0.65 | 0.67 | 1.24 | 0.60 | 1.21 | 0.91 | 0.80 |
| *Hoxb9* | 1.10 | 1.28 | 1.09 | 1.80 | 0.88 | 0.53 | 0.57 | 1.45 | 1.20 | 1.84 | 1.18 | 1.09 |
| *Hoxb9ex* | 0.56 | 1.07 | 0.51 | 1.22 | 1.09 | 0.22 | 0.69 | 0.96 | 0.86 | 1.25 | 1.47 | 0.55 |
| *Gapdh* | 1.05 | 1.25 | 1.18 | 0.90 | 1.30 | 0.80 | 0.87 | 1.13 | 1.16 | 0.88 | 1.35 | 0.65 |
|  |  |  |  |  |  |  |  |  |  |  |  |  |
| *Pou5f1* |  |  |  |  |  |  |  |  |  |  |  |  |
| *Nanog* |  |  |  |  |  |  |  |  |  |  |  |  |
| *Cdx2* |  |  |  |  |  |  |  |  |  |  |  |  |
| *Gapdh* |  |  |  |  |  |  |  |  |  |  |  |  |
|  |  |  |  |  |  |  |  |  |  |  |  |  |
