# Supplementary material for: Transcription-Independent Heritability of Induced Histone Modifications in the Mouse Preimplantation Embryo
Source: PLoS One. 2009 Jun 30;4(6):e6086. doi: 10.1371/journal.pone.0006086 (PMC2698989; doi:10.1371/journal.pone.0006086)
Supplement: Table S5 — Primers used for CChIP analysis (0.03 MB DOC) [file pone.0006086.s008.doc]

## Supplementary Table S5

*Primers used for CChIP Analysis*

| Gene Name | Forward 5’-3’ | Reverse 5’-3’ | Tm |
| --- | --- | --- | --- |
|  |  |  |  |
| *Nanog* | tcacactgacatgagtgtgg | tctgtgcagagcatctcagt | 58 |
| *Cdx2* | aaatcgtgtttctgggg | ccttacgtgattaacgagtg | 55 |
| *Gapdh* | tgtgccaagcacttgtataac | tatgtctgaccagaggagagca | 60 |
| *Pou5f1* | ctgtaaggacaggccgagag | caggaggccttcattttcaa | 60 |
| *Hoxb1* | agatggatgggctcagagtg | taggaaggggctagggagtg | 55 |
| *Hoxb9* | ctcgcccgattgatttatgt | caccccctgctcaacttct | 58 |
| *Hoxb9* | gcacgcccgagtacagtttg | cctctctttgtcctcgcttcct | 58 |

Primer locations across the genes listed can be found in O’Neill, L.P. et al. Nat Genet. 38, 835-841 (2006) and Chambeyron S. & Bickmore W.A. Genes Dev. 18, 1119-30 (2004)
