# Supplementary material for: Transcription-Independent Heritability of Induced Histone Modifications in the Mouse Preimplantation Embryo
Source: PLoS One. 2009 Jun 30;4(6):e6086. doi: 10.1371/journal.pone.0006086 (PMC2698989; doi:10.1371/journal.pone.0006086)
Supplement: Table S6 — Primers used for expression analysis (0.03 MB DOC) [file pone.0006086.s009.doc]

**Supplementary Table S6**

*Primers used for Expression Analysis*

| Gene name | Forward 5’-3’ | Reverse 5’-3’ | Tm |
| --- | --- | --- | --- |
|  |  |  |  |
| *Gapdh* | tgatgacatcaagaaggtggtgaag | tccttggaggccatgtaggccat | 60 |
| *Hoxb1* | ccatatcctccgccgcag | cggactggtcagaggcatc | 62 |
| *Hoxb9* | cagggaggctgtcctgtctaatc | cttctctagctccagcgtctgg | 62 |
| *Pou5f1* | ccaatcagcttgggctagag | cctgggaaaggtgtcctgta | 60 |
| *Nanog* | gcaagcggtggcagaaaaac | gcaatggatgctgggatactca | 60 |
| *Cdx2* | ggaagccaagtgaaaacca | ccagctcacttttcctccg | 60 |
| *ActB* | tgacagggatgcagaaggaga | gctggaaggtggacagtgag | 60 |

Primer locations across the genes listed can be found in O’Neill, L.P. et al. Nat Genet. 38, 835-841 (2006) and Chambeyron S. & Bickmore W.A. Genes Dev. 18, 1119-30 (2004)
